# Does monosodium glutamate really cause headache? : a systematic review of human studies

**DOI:** 10.1186/s10194-016-0639-4

**Published:** 2016-05-17

**Authors:** Yoko Obayashi, Yoichi Nagamura

**Affiliations:** International Glutamate Technical Committee (IGTC), Avenue Jules Bordet 142, B-1140 Brussels, Belgium; Faculty of Health Science, Department of Clinical Nurition, Suzuka University of Medical Science, 1001-1 Kishioka-cho, Suzuka-city, Mie 510-0293 Japan

**Keywords:** Monosodium glutamate (MSG), Headache, International classification of headache disorders (ICHD), Systematic review, Human study, Chinese restaurant syndrome (CRS)

## Abstract

Although monosodium glutamate (MSG) is classified as a causative substance of headache in the International Classification of Headache Disorders 3rd edition (ICHD-III beta), there is no literature in which causal relationship between MSG and headache was comprehensively reviewed. We performed systematic review of human studies which include the incidence of headache after an oral administration of MSG. An analysis was made by separating the human studies with MSG administration with or without food, because of the significant difference of kinetics of glutamate between those conditions (Am J Clin Nutr 37:194–200, 1983; J Nutr 130:1002S–1004S, 2000) and there are some papers which report the difference of the manifestation of symptoms after MSG ingestion with or without food (Food Chem Toxicol 31:1019–1035, 1993; J Nutr 125:2891S-2906S, 1995). Of five papers including six studies with food, none showed a significant difference in the incidence of headache except for the female group in one study. Of five papers including seven studies without food, four studies showed a significant difference. Many of the studies involved administration of MSG in solution at high concentrations (>2 %). Since the distinctive MSG is readily identified at such concentrations, these studies were thought not to be properly blinded. Because of the absence of proper blinding, and the inconsistency of the findings, we conclude that further studies are required to evaluate whether or not a causal relationship exists between MSG ingestion and headache.

## Background

Monosodium glutamate is used worldwide as a flavor enhancer [1–4]. So called Chinese restaurant Syndrome (CRS) was first reported by Kwok in 1968 [1]. He reported that after consumption of Chinese dishes, some transient subjective symptoms occurred such as numbness, general weakness, palpitation, etc. Although many human studies were conducted afterwards to determine if a causal relationship occurs between MSG and this symptom complex, the results were inconsistent. Headache was reported to be one of this symptom complex.

The safety of glutamic acid and its salts as flavor enhancers was evaluated by the Joint FAO/WHO Expert Committee on Food Additives in 1971 [2], 1974 [3], and 1987 [4] and it allocated an “acceptable daily intake (ADI) not specified” based on the conclusion that the total intake of glutamate arising from their use at levels necessary to achieve the desired technological effect and from their acceptable background in food do not represent a hazard to health.

In this review, we report the results of a systematic review of available human studies of MSG, focusing on the causal relationship between MSG intake and headache by separate analysis of the studies with MSG administration with or without food. We also discuss the scientific validation of ICHD-III beta on MSG [5], based on our review of the studies cited.

## Review

### Methods

Since most of the human studies that include data on the headache incidence focus on symptom complex and do not specifically focus on headache, search condition was optimized to obtain as many human study data with MSG administration as possible.

The literature search was conducted on Medline and FSTA. Initially, we searched for titles that includes any of the following terms: “monosodium glutamate”, “MSG”, “monosodium L-glutamate”, “sodium glutamate”, and “sodium L-glutamate”. In case of Medline, the search was narrowed down by specifying article type as “clinical trial”. The above search result was further narrowed down by the following conditions; (1) the paper is written in English, (2) the paper is a human study with the administration of MSG using healthy adults, (3) the incidence of headache is shown, and (4) a statistical analysis was performed on the incidence of headache, or the paper includes sufficient data with which to perform statistical analysis.

## Results and discussion

### Result of the literature search

The literature search identified eight human studies using Medline and two human studies using FSTA that met our inclusion criteria. Of these ten papers, five papers were the human studies with the MSG administration with food and the dose of MSG was 1.5 – 3.15 g [6–10]. The other five papers were the studies with the MSG administration without food and the dose of MSG was 1.25 – 12 g [11–15]. The outline of these studies is shown in Tables 1 and 2. ICHD-III beta referenced five papers concerning MSG in section 8.1 [6, 12, 16–18]. The outline of these papers is shown in Tables 3 and 4.

**Table 1:** Human studies of MSG with food

First author	Vehicle for MSG administration	Protocol	Number of subjects	Incidence of headache	Statistical difference
Prawirohardjono W (2000) [7]	Opaque capsule	(1)In the morning, after fasting for 10 h, subjects ingested three capsules containing MSG(0.5 g MSG & 0.5 g lactose or 1.0 g MSG) or placebo(1.0 g lactose). (2)A standardized breakfast was provided and consumed immediately after capsule ingestion.	52 healthy volunteers (Indonesians) (Mean age 29.6 ± 6.5y, mean mass 53.4 ± 7.4 kg, mean height 159.9 ± 7.7 cm) *No indication about gender.	Placebo : 3 1.5 g MSG : 4 3.0 g MSG : 2	No difference
Tarasoff L (1993) [6]	Capsule	Fasting condition On first 3 days, 6 capsules immediately followed by breakfast.	71 healthy volunteers (female:41, mean age:30.7) *Mainly Caucasians	Placebo (gelatin powder): 1 1.5 g MSG: 0 3.0 g MSG: 0	No difference
	Specially formulated drinks	On remaining 2 days, 300 ml soft drink immediately followed by breakfast.		Placebo (drink): 0 3.15 g MSG (drink): 0	No difference
Tanphaichitr V (1983) [8]	Boiled rice with pork	Menu A-D(w/o MSG), E(added 3 g MSG) were serve as breakfast on Day 1–5 by this order.	50 adults (male:25, female:25)	Menu A(w/o MSG):4 Menu B(w/o MSG):0 Menu C(w/o MSG):2 Menu D(w/o MSG):2 Menu E(3 g MSG):0	No difference
Zanda G (1973) [9]	Beef bouillon	3 g MSG(placebo: no MSG, no substitute) in 150 ml beef bouillon followed by other dishes. [First session] Some subjects at random received MSG. [Second session(2 days later)] Opposite to the first session	73 healthy volunteers (male:38, female:35, mean age:25, 17–76y)	Control(male):1 Control(female):1 3 g MSG (male):1 3 g MSG (female):6* (*P* < 0.05) Responded to both (male):2 Responded to both (female):2	Only MSG-treated women had a significantly higher incidence of headache than control.
		Blood pressure and pulse rate were also recorded.			
Morselli PL (1970) [10]	Beef broth	3 g MSG in 150 ml beef broth followed by other dishes(meat, vegetables, fruit). [First session] MSG:8 sub., control:16 sub. [Second session(2 days later)] Opposite to the first session	24 healthy volunteers (male:17, female:7)	Control: 1 3 g MSG: 2	No difference

*; Statistically significant difference was found between placebo and MSG group (*P* < 0.05)

**Table 2:** Human studies of MSG without food

Researcher	Vehicle for MSG administration	Protocol	Number of subjects	Incidence of headache	Statistical difference
Geha RS (2000) [11]	200 ml of citrus-flavored beverage	[Protocol A] 5 g MSG and placebo (0 g MSG) on separate day (day 1 & 2)	130 self-reported MSG-reactive volunteers (female:84, male:46)	[Frequency] Placebo: 0.28 5 g MSG: 0.54 (*P* < 0.005)**	Significant difference
Yang WH (1997) [12]	200 ml of a strongly citrus-tasting beverage sweetened by sucrose.	(1) 5 g MSG or placebo(0 g MSG) (empty stomach)	61 subjects self-identified MSG-sensitive people (male:15, white:59, black:1, oriental:1)	Placebo: 24 5 g MSG: 23	No difference
		(2) placebo(0 g MSG, no substitute), 1.25, 2.5, 5 g MSG in random sequence.	36 subjects Subjects who responded to either of MSG or placebo in study(1) (not both or not neither). No indication about gender.	Placebo: 9 1.25 g MSG: 11 2.5 g MSG:16(*P* < 0.04)* 5 g MSG: 18(*P* < 0.023)*	1.25 g: no difference 2.5, 5 g:significant difference
Shimada A (2013) [13]	400 ml Sugar-free lemon soda	MSG (150 mg/kg = 9 g/60 kg) for 5 consecutive days (in the week) and NaCl (24 mg/kg) in the other week in randomized sequence.	14 healthy adults (female:9, male:5)	Placebo: 2 150 mg/kg MSG(=9 g/60 kg): 8* (P = 0.041)	Significant difference
Baad-Hansen L (2010) [14]	400 ml Sugar-free soda	NaCl (24 mg/kg), MSG (75 or 150 mg/kg =6 or 9 g/60 kg) in random sequence.	14 healthy men	Placebo: 0 75 mg/kg MSG(=4.5 g/60 kg): 27* (P = 0.045, vs placebo and 150 mg/kg MSG) 150 mg/kg MSG (=9 g/60 kg): 7	75 mg/kg: significant difference 150 mg/kg: no difference
Rosenblum I (1971) [15]	100 ml tap water or chicken stock	15 h after the last meal. (Group I–IV)5 g MSG in tap water(49 subjects) or chicken stock(49 sub.). (Group V)1.7 g NaCl in chicken stock(24 sub.) (Group VI) chicken stock(25 sub.).	99 male volunteers, 21–59 years old.	[Frequency] Placebo(Group V, VI):8 % 5 g MSG(Group I–IV):17 %	No difference
	100 ml chicken stock	(Group VII) 8 g(6 sub.) MSG in chicken stock, 2.8 g NaCl in chicken stock(5 sub.). (Group VIII) 12 g MSG in chicken stock(5 sub.), 4.2 g NaCl in chicken stock (5 sub).	11 people Chosen from the original 99 subjects, based on the results of the test above. One-half reported multiple complaints on the questionnaire while the other reported no complaints.	Placebo(NaCl2.8 g):2 8 g MSG(VII):3 Placebo(NaCl4.2 g):0 12 g MSG(VIII):2	No difference

*; Statistically significant difference was found between placebo and MSG group (*P* < 0.05)

**; Statistically significant difference was found between placebo and MSG group (*P* < 0.01)

**Table 3:** Human studies of MSG which were referenced by ICHD-III beta

First author	study type	Number of subjects	MSG administration	with (w) or without(w/o) food	Statistical analysis in the paper	statistical difference
Tarasoff L (1993) [6]	human study	71	capsule	w	performed	No difference
			3.15 g MSG/300 ml soft drink	w	performed	No difference
Yang WH (1997) [12]	human study	61(self-identified MSG sensitive)	5 g MSG/200 ml strongly citrus-tasting beverage	w/o	performed	No difference
		36(self-identified MSG sensitive)	1.25–5 g MSG/200 ml strongly citrus-tasting beverage	w/o	performed	2.5, 5 g: Significant difference
Gore M (1980) [16]	human study	55	1.5–6 g MSG/150 ml tap water	w/o	not performed	No difference (Fisher test)
Kenny RA (1972) [17]	human study	77	5 g MSG/150 ml tomato juice	w/o	not performed	No difference (Fisher test)
		22	1–5 g MSG/ 150 ml tomato juice or water	w/o	not performed	cannot analyze
Merrit JE (1990) [18]	in vitro

**Table 4:** Human studies of MSG which were referenced by ICHD-III beta but were not complied with the criteria for the systematic review

First author	Vehicle for MSG administration	Protocol	Number of subjects	Incidence of headache	Statistical difference
Gore M (1980) [16]	150 ml cold tap water	After an overnight fast, subjects ingested, on different days, 1.5, 3, 6 g MSG and three paired placebo materials, the order of each pair being randomized. (There was no indication about the content "three paired placebo materials")	30 men, 25 women	[Total number of positive responses by 3 doses] MSG: 8 episodes (7 subjects), Placebo: 2 episodes (2 subjects)	No difference (Fisher test) Statistical analysis in each symptom was not performed in the paper.
Kenny RA (1972) [17]	150 ml tomato juice	(Phase 1) 2 h after breakfast, 5 g MSG on1 day and 0.8 g NaCl on the other 2 days. Breakfast type: (1)no breakfast (2)liquids (milk, coffee, juice) or instant breakfast (3)largely of carbohydrate(cereal, toast, etc.) (4)containing protein (eggs, ham, etc.)	(Phase 1) 77 subjects	[Number of positive responses] MSG : 4, Placebo : 2	No difference (Fisher test) Statistical analysis was not performed in the paper.
	150 ml tomato juice (J) or water (W)	(Phase 2) JP1 : 0.8 g NaCl, WP: 0.7 g NaCl, WM5 : 5 g MSG + 0.2 g NaCl, JM1 : 1 g MSG, JM2 : 2 g MSG, JM3 : 3 g MSG, JM4 : 4 g MSG, JP2 : 0.8 g NaCl	(Phase 2) 22 out of 25 subjects, who reacted only to MSG on phase 1 study	[Number of positive responses / subjects] JM1 : 1 /15, JM2 : 2/13, JM3 : 4/13, JM4 : 5/14, WM5 : 8/17	Since the incidence of placebo group (JP1, WP, JP2) is not shown, statistical analysis cannot be done.

### Blind integrity

The normal content of added MSG in food is 0.2–0.8 % for appropriate taste. Kenny reported that the subjects responded favorably in terms of taste and palatability of tomato juice containing 1 g MSG in 150 ml and less favorably when larger quantities (>2 g) were given [17]. In addition, Gore reported that there were statistically significant difference between the taste and after taste of 1–4 % MSG dissolved in water compared with placebo solution [16]. It is also reported that high dose of MSG causes various adverse gastrointestinal reactions, leading to vomiting at very high dose [19]. Taken together, it can be assumed that the beverage containing 1.3 % MSG (2 g/150 ml-) or more should be distinguishable from placebo beverage and have an unfavorable taste.

Therefore, we analyzed the human studies, especially paying attention to the blind integrity and validity of the data.

### Human studies with MSG administration with food

Regarding the studies with the MSG administration with food, five papers which include six studies were found (Table 1).

On the study reported by Praworphardjono [7] and at the first stage of Tarasoff’s study [6], 1.5, 3.0 g MSG or placebo in capsules was administered in fasted conditions, followed by the ingestion of a standardized breakfast. At the second stage of Tafasoff”s study, the subjects ingested a specially formulated soft drink which included 3.15 g MSG or placebo, and then consumed the standardized breakfast. Although at this second stage, some subjects might be able to distinguish placebo and MSG beverage, they analyzed only the data from 61 subjects, who reported no after-taste, out of 71 subjects. In all studies by Praworphardjono and Tarasoff, no difference of incidence of headache between placebo and MSG administration was found and the authors concluded that rigorous and realistic scientific evidence linking CRS to MSG could not be found.

Tanphaichitr investigated the incidence of unpleasant symptoms after ingestion of a breakfast containing added MSG or no added MSG [8]. In the first experiment, using ten subjects, a menu that masked the taste of MSG was identified. Four menus without MSG and one menu with 3 g MSG were served to 50 subjects as a breakfast on day 1–5 one-by-one. No one served with the menu containing MSG had a headache and the author concluded that the addition of MSG did not cause significant difference in unpleasant symptoms of CRS from those on menu without MSG.

Thus, those three studies above can be thought properly blinded, but the following two studies reported by Zanda [9] and Morselli [10] might not ensure the sufficient blind integrity. In both studies, 3 g MSG in 150 ml beef broth (2 %) was administered followed by other dishes. No significant difference in the incidence of CRS symptoms was found in both studies, with the exception of headache occurrence among females in Zanda’s study: an incidence in 6 subjects was observed on the MSG day, but only 1 on the placebo day (*P* < 0.05). Clinically meaningful differences in objective indices, such as arterial blood pressure and pulse rate, were not observed. In total 73 subjects (38 males, 35 females) joined this study. We note that the number of migrainers is higher in females than in males (France; male 6.35 %, female 15.7 %, USA; male 6.6 %, female 19.2 %) [20], and unpleasant taste and unfavorable sensation caused by high dose of MSG may become a stimulant to trigger headache on migrainers especially when they are fasted. Placebo effect by distinguishable taste also should not be ignored.

In the study by Morselli, they concluded that no difference in all CRS symptoms including headache between the subjects given MSG and control broth.

### Human studies with MSG administration without food

Regarding the studies of MSG administration without food, we found five papers containing seven studies (Table 2). In all of those studies, MSG dissolved in beverage or soup at relatively high concentrations (1.125 – 12 %) was administered to the subjects.

A statistical difference of incidence of headache was reported in four out of seven studies. In two of those four studies reported by Geha [11] and Yang [12], self-identified MSG sensitive subjects were recruited and 200 ml of a citrus-flavored beverage with or without MSG was administered to 130 and 36 subjects, respectively. In Geha’s study, the dose of MSG was 5 g (2.5 %) and the significant difference of incidence of headache in MSG group was found. Yang’s study was composed of two studies and the first study did not show statistical difference, despite of a large amount of MSG, i.e. 5 g (2.5 %), ingested. However, at the second stage, in which the subjects reacted to either of MSG or placebo at the first stage joined, a significant difference in the incidence of headache at the dose of 2.5 and 5.0 g MSG (1.25 and 2.5 %) and the dose dependency was reported.

Among three other studies using subjects who were not restricted to self-identified MSG sensitive subjects, a significant difference in the incidence of headache was found in two studies, both of which originated in the same laboratory in Denmark [13, 14]. In the first study reported by Baad-Hansen, 400 ml sugar-free soda containing MSG (75 or 150 mg/kg) or NaCl (24 mg/kg) was administered to 14 healthy men [14]. These doses correspond to 4.5 g (1.125 %) and 9.0 g (2.25 %) /400 ml・60 kg b.w., respectively. A significant difference in the incidence of headache was observed at the 75 mg/kg dose of MSG, but not at the 150 mg/kg dose compared to the NaCl placebo. In the second study reported by Shimada, the protocol and the number of subjects were the same as in the first study, except that the number of days in one session was increased from 1 to 5 days to amplify the incidence and the MSG dose was 150 mg/kg, along with the placebo 24 mg/kg NaCl [13]. During one session, either of MSG or NaCl was administered. The 400 ml volume and the high dose of MSG, especially 150 mg/kg, are thought to be sufficient to cause gastrointestinal unpleasant sensation attributable to unfavorable taste and high osmotic pressure. The other issue of these studies are that the content of sodium in placebo (24 mg/kg) corresponds to 75 mg/kg MSG and half of 150 mg/kg MSG. It means that the saltiness and osmotic pressure caused by placebo solution was much less than MSG solution of 150 mg/kg dose. In addition, the number of 14 subjects is too small to permit any conclusion regarding large population.

On the other hand, in Rosenblum’s study using 99 male subjects, 5, 8, 12 g MSG (5, 8, 12 %) and osmotically equivalent dose of NaCl dissolved in 100 ml tap water or chicken stock was administered [15]. A significant difference in the incidence of headache was not observed at any dose.

Thus, the results of those studies are inconsistent and it is difficult to conclude whether MSG ingestion without food causes headache. The effect of blind integrity seems to be more influential to self-identified MSG-sensitive subjects.

### Human studies which were cited by ICHD-III beta

Among the five papers cited (Table 3), two complied with our search criteria, and are discussed above [2, 11]. The other two human study papers which did not comply with our search criteria were authored by Gore and Kenny, respectively (Table 4) [16, 17]. Gore’s paper does not contain the term meaning “monosodium-L-glutamate” in the title, and in Kenny’s paper, the statistical analysis could not be performed due to a lack of placebo data. Statistical analysis was not performed in either papers. The remaining one paper by Meritt was an in vitro study [18].

Gore et al. reported the study using 55 subjects. They ingested 1.5, 3, 6 g MSG and three paired placebo materials dissolved in 150 ml cold tap water on different days [16]. The incidence of headache was shown as a total number of incidences by three doses, which did not show statistical difference according to our analysis.

In the study reported by Kenny et al., MSG-reactors were screened from 77 subjects at the first study by the administration of 150 ml tomato juice with 5 g MSG or 0.8 g NaCl [17]. According to our analysis, a statistical difference in the incidence of headache was not observed in the first study. Twenty-two MSG-reactors, who suffered from any symptoms in the first study, formed test groups in the second study and were administered 1–5 g MSG dissolved in 150 ml tomato juice or water. We could not perform a statistical analysis on the results, due to a lack of placebo data.

Among above-mentioned four papers on human studies, consisting of six studies, a statistical difference was found only in one study reported at the second stage of Yang’s study, which is aforementioned.

The in vitro study authored by Merrit focused on a direct effect of MSG on contraction and relaxation of rabbit aorta. However, we should pay attention to the little impact of MSG ingestion on plasma glutamate level, especially when MSG is ingested with food as a flavor enhancer [21]. Tsai et al. reported the circadian variation of plasma glutamate level when the meals added 100 mg/kg MSG (15, 40, 45 mg/kg to breakfast, lunch and dinner) were given to healthy adult men. It ranged between 33 and 48 μmol/l on days without added MSG, and 32 and 53 μmol/l on days with added MSG [22]. Although 100 mg/kg MSG (6 g MSG/60 kg bw) is much larger dose than the average MSG intake from food (estimated intake of added glutamate is c.a. 0.4 g in Europe and 1.2–1.7 g in Asian countries [23]), about 10 % change of circadian variation can be regarded as being within the rage of daily variation.

Taken together, it is difficult to argue from this group of studies that MSG causes headache.

## Conclusion

Among human studies with the MSG administration with food, significant difference of headache incidence was not found at the dose of 1.5 and 3.0 g in capsule, 3.15 g/300 ml beverage, 3.0 g in boiled rice with pork, and 3.0 g/150 ml beef broth. The significant difference was found only in female administered 3.0 g MSG/150 ml (2.0 %) beef bouillon but not in male.

In all the studies with MSG administration without food, MSG was administered, being dissolved in beverages or soup at relatively high concentrations (1.125–12 %). In those studies, significant difference of headache incidence was found at the dose of 2.5 g/200 ml, 5.0 g/200 ml, 6 g/400 ml・60 kg bw, 9 g/400 ml・60 kg bw, but not found at 1.25 g/200 ml, 5.0 g/200 ml, 9 g/400 ml・60 kg bw, 5.0 g/100 ml, 8.0 g/100 ml, 12.0 g/100 ml.

We should pay attention to the blind integrity of the human studies where high dose of MSG was administered in solution, especially focusing on the distinguishable and unpleasant taste of MSG solutions at 1.3 % (2 g/150 ml) or more and the gastrointestinal discomfort caused by high dose of MSG. These events may influence the occurrence of headache quite strongly especially in case of migrainers and the subjects who believe they are MSG-sensitive.

From the fact that the results of the human studies are not consistent and it is assumed that most studies using beverages as a vehicle are not properly blinded, we suggest that a causal relationship between MSG and headache has not been proven. In addition, statistically significant differences in the incidence of headache were not observed when MSG was administered with food, except in one case of the female group where the blind integrity was questionable. It would seem premature to conclude that the MSG present in food causes headache.

## Abbreviations

MSG: monosodium glutamate
ICHD: international classification of headache disorders
CRS: Chinese restaurant syndrome
FSTA: food science technology abstracts
ADI: acceptable daily intake

## References

[1] Kwok RH (1968). Chinese-restaurant syndrome. N Engl J Med.

[2] FAO/WHO (1971) Evaluation of food additives: specifications for the identity and purity of food additives and their toxicological evaluation; some extraction solvents and certain other substances; and a review of the technological efficiency of some antimicrobial agents. 14th Report of the Joint FAO/WHO Expert Committee on Food Additives. FAO Nutrition Meetings Report Series no. 48, WHO Technical Report Series no. 462

[3] FAO/WHO (1974) Toxicological evaluation of certain food additives with a review of general principles and of specifications. 17th Report of the Joint FAO/WHO Expert Committee on Food Additives. FAO Nutrition Meetings Report Series no. 53, WHO Technical Report Series no. 539

[4] Joint FAO/WHO Expert Committee on Food Additives (1988). L-glutamic acid and its ammonium, calcium, monosodium and potassium salts. In: Toxicological Evaluation of Certain Food Additives and Contaminants.

[5] Headache Classification Committee of the International Headache Society (IHS) (2013). The International Classification of Headache Disorders, 3rd edition (beta version). Cephalalgia.

[6] Tarasoff L, Kelly MF (1993). Monosodium L-glutamate: a double-blind study and review. Food Chem Toxicol.

[7] Prawirohardjono W, Dwiprahasto I, Astuti I, Hadiwandowo S, Kristin E, Muhammad M, Kelly MF (2000). The administration to Indonesians of monosodium L-glutamate in Indonesian foods: an assessment of adverse reactions in a randomized double-blind, crossover, placebo-controlled study. J Nutr.

[8] Tanphaichitr V, Srianujata S, Pothisiri P, Sammasut R, Kulapongse S (1983). Post prandial responses to Thai foods with and without added monosodium L-glutamate. Nutr Rep Int.

[9] Zanda G, Franciosi P, Tognoni G, Rizzo M, Standen SM, Morselli PL, Garattini S (1973). A double blind study on the effects of monosodium glutamate in man. Biomedicine.

[10] Morselli PL, Garattini S (1970). Monosodium glutamate and the Chinese restaurant syndrome. Nature (Lond.).

[11] Geha RS, Beiser A, Ren C, Patterson R, Greenberger PA, Grammer LC, Ditto AM, Harris KE, Shaughnessy MA, Yarnold PR, Corren J, Saxon A (2000). Multicenter, double-blind, placebo-controlled, multiple-challenge evaluation of reported reactions to monosodium glutamate. J Allergy Clin Immunol.

[12] Yang WH, Drouin MA, Herbert M, Mao Y, Karsh J (1997). The monosodium glutamate symptom complex: assessment in a double-blind, placebo-controlled, randomized study. J Allergy Clin Immunol.

[13] Shimada A, Cairns BE, Vad N, Ulriksen K, Pedersen AM, Svensson P, Baad-Hansen L (2013). Headache and mechanical sensitization of human pericranial muscles after repeated intake of monosodium glutamate (MSG). J Headache Pain.

[14] Baad-Hansen L, Cairns B, Ernberg M, Svensson P (2010). Effect of systemic monosodium glutamate (MSG) on headache and pericranial muscle sensitivity. Cephalalgia.

[15] Rosenblum I, Bradley JD, Coulston F (1971). Single and double blind studies with oral monosodium glutamate in man. Toxicol Appl Pharmacol.

[16] Gore ME, Salmon PR (1980). Chinese restaurant syndrome: fact or fiction?. Lancet.

[17] Kenney RA, Tidball CS (1972). Human susceptibility to oral monosodium L-glutamate. Am J Clin Nutr.

[18] Merritt JE, Williams PB (2005). Vasospasm Contributes to Monosodium Glutamate-lnduced Headache. Headache.

[19] Bizzi A, Veneroni E, Salmona M, Garattini S (1977). Kinetics of monosodium glutamate in relation to its neurotoxicity. Toxicol Lett.

[20] Lj S, Hagen K, Jensen R, Katsarava Z, Lipton R, Scher A, Steiner T, Zwart JA (2007). The global burden of headache: a documentation of headache prevalence and disability worldwide. Cephalalgia.

[21] Stegink LD, Baker GL, Filer LJ (1983). Modulating effect of sustagen on plasma glutamate concentration in humans ingesting monosodium L-glutamate. Am. J. Clin. Nutr.

[22] Tsai PJ, Huang PC (2000). Circadian variations in plasma and erythrocyte glutamate concentrations in adult men consuming a diet with and without added monosodium glutamate. J Nutr.

[23] Beyreuther K, Biesalski HK, Fernstrom JD, Grimm P, Hammes WP, Heinemann U, Kempski O, Stehle P, Steinhart H, Walker R (2007). Consensus meeting: monosodium glutamate - an update. Eur J Clin Nutr.

